# Quantitative proteomic studies addressing unmet clinical needs in sarcoma

**DOI:** 10.3389/fonc.2023.1126736

**Published:** 2023-05-01

**Authors:** Elizabeth A. Connolly, Peter S. Grimison, Lisa G. Horvath, Phillip J. Robinson, Roger R. Reddel

**Affiliations:** ^1^ ProCan^®^, Children’s Medical Research Institute, Faculty of Medicine and Health, The University of Sydney, Westmead, NSW, Australia; ^2^ Department of Medical Oncology, Chris O’Brien Lifehouse, Sydney, NSW, Australia; ^3^ Faculty of Medicine and Health, The University of Sydney, Sydney, NSW, Australia

**Keywords:** sarcoma, proteomics, biomarkers, drug discovery, mass spectrometry

## Abstract

Sarcoma is a rare and complex disease comprising over 80 malignant subtypes that is frequently characterized by poor prognosis. Challenges in clinical management include uncertainties in diagnosis and disease classification, limited prognostic and predictive biomarkers, incompletely understood disease heterogeneity among and within subtypes, lack of effective treatment options, and limited progress in identifying new drug targets and novel therapeutics. Proteomics refers to the study of the entire complement of proteins expressed in specific cells or tissues. Advances in proteomics have included the development of quantitative mass spectrometry (MS)-based technologies which enable analysis of large numbers of proteins with relatively high throughput, enabling proteomics to be studied on a scale that has not previously been possible. Cellular function is determined by the levels of various proteins and their interactions, so proteomics offers the possibility of new insights into cancer biology. Sarcoma proteomics therefore has the potential to address some of the key current challenges described above, but it is still in its infancy. This review covers key quantitative proteomic sarcoma studies with findings that pertain to clinical utility. Proteomic methodologies that have been applied to human sarcoma research are briefly described, including recent advances in MS-based proteomic technology. We highlight studies that illustrate how proteomics may aid diagnosis and improve disease classification by distinguishing sarcoma histologies and identify distinct profiles within histological subtypes which may aid understanding of disease heterogeneity. We also review studies where proteomics has been applied to identify prognostic, predictive and therapeutic biomarkers. These studies traverse a range of histological subtypes including chordoma, Ewing sarcoma, gastrointestinal stromal tumors, leiomyosarcoma, liposarcoma, malignant peripheral nerve sheath tumors, myxofibrosarcoma, rhabdomyosarcoma, synovial sarcoma, osteosarcoma, and undifferentiated pleomorphic sarcoma. Critical questions and unmet needs in sarcoma which can potentially be addressed with proteomics are outlined.

## 1 Background

Sarcomas are rare mesenchymal malignancies of bone and soft tissue that are frequently characterized by poor prognosis (1). They account for approximately 1% of adult malignancies and up to 15% of childhood cancers (1–3). There are more than 80 histological subtypes of sarcoma and approximately 20% of all sarcomas are defined as ‘ultra-rare’ with an incidence of less than 1 in 1,000,000 (4). The heterogeneity of sarcomas and the rarity of many subtypes makes their study difficult, and their clinical management challenging.

Classification of sarcomas is complicated not only by the heterogeneity in histological findings among subtypes, but also within subtypes. Many sarcomas, such as leiomyosarcoma (LMS) or undifferentiated pleomorphic sarcoma (UPS), do not have definitive diagnostic markers. Several sarcomas are characterized by unique molecular findings, which can be diagnostic, however the equipment and expertise to conduct the molecular tests required are not routinely available in many laboratories (5, 6). The challenge of histopathological diagnosis in sarcoma is exemplified in studies where sarcoma pathology has been subjected to expert second review, with major discrepancies in histological subtype diagnosis being identified in up to 25% of cases (6, 7).

Sarcomas can have a wide variety of clinical presentations and outcomes, although many subtypes are associated with aggressive natural histories and poor prognosis. Despite optimal resection and the use of neo-adjuvant and adjuvant therapies, localized soft tissue and bone sarcomas demonstrate high rates of distant relapse (8–10), and the clinical trial evidence that neo-adjuvant or adjuvant chemotherapy reduces the risk of relapse of soft tissue sarcoma (STS) is inconclusive (11–13). It is currently not possible to predict with sufficient accuracy which individuals are at greatest risk of relapse and need more intensive or different therapies. The prognosis of advanced disease is typically poor, with median survival less than two years in most STS and bone sarcomas even in adolescents and young adults (14–17). Systemic treatment options in advanced disease are limited, with low response rates and short-duration responses (14, 18–20).

Prognostic biomarkers currently in routine clinical use are largely clinico-pathological. Examples include degree of histological necrosis in osteosarcoma (21), and clinicopathological nomograms in rhabdomyosarcoma (RMS) and other STS that include primary lesion size, grade and histological subtype (8, 22–25). Genomic biomarkers, including ‘CINSARC’ which is a validated prognostic 67-gene expression signature for clinical outcome in sarcoma, have been developed but have not been integrated into routine clinical practice to date (26–28). There are a limited number of predictive biomarkers in routine clinical use in soft tissue or bone sarcoma. In RMS, negative prognostic clinicopathological variables classifying patients as ‘high risk’ were found to be predictive of benefit from maintenance chemotherapy with vinorelbine and cyclophosphamide (29). Prognostic clinicopathological biomarkers, however, have not been shown to be predictive of adjuvant treatment efficacy in STS clinical trials nor has histological necrosis in osteosarcoma for maintenance or intensified post-operative therapy (10, 30).

This review describes the current and potential contribution of studying proteins in sarcoma. The utility of studying the expression of *specific* proteins has been firmly established in oncology, but for a limited number of applications. Positive immunohistochemical protein expression, for example, plays a key role in the diagnostic work up of sarcomas. The absence of cytokeratin staining can help to differentiate sarcomas from carcinomas and expression of endothelial marker CD31 is used to identify vascular soft tissue tumors (31). Expression of myogenic proteins desmin, MYOD1 and myogenin define RMS and are used to differentiate RMS from other round cell tumors (32). Protein-based tumor markers, such as carcinoembryonic antigen in colorectal cancer, are used for disease monitoring and proteins may guide anti-cancer treatment (33). Altered mismatch repair protein expression, for example, may predict response to immunotherapy (34, 35). Proteins also form the target that many molecularly targeted cancer drugs interact with. Examples in sarcoma include anaplastic lymphoma kinase (ALK) receptor inhibition with crizotinib in ALK-rearranged inflammatory myofibroblastic tumors and platelet-derived growth factor receptor alpha (PDGFRA) and KIT receptor inhibition in gastrointestinal stromal tumors (GIST) with a panel of agents including imatinib, sunitinib, regorafenib, ripretinib and avapretinib (36–42). Multi-tyrosine kinase inhibitors such as cediranib, pazopanib, and cabozantinib, which inhibit multiple receptors including vascular endothelial growth factor (VEGF) receptors, have demonstrated clinical efficacy in alveolar soft part sarcoma, non-lipogenic STS, and bone sarcomas respectively (20, 43, 44).

Proteomics refers to the study of *all* detectable proteins expressed in specific cells or tissues: their abundance, activity and interactions. Proteins play a key role in the functioning of all cells and tissues including cancers. Proteomics has the potential to improve understanding of the biology and disease behavior of sarcoma as an entity and of sarcoma subtypes, and identify prognostic and predictive biomarkers of disease outcomes including treatment response that could better inform treatment decisions and improve survival of patients with sarcoma. Proteomics could also inform therapeutic target discovery and development of novel therapeutics for localized and advanced disease.

Proteins represent the dynamic downstream products of the more than 20,000 genes comprising the genome following DNA transcription, RNA translation and post translational modifications. Genomic findings alone have not fully accounted for the complexities of cancer biology. Because protein expression levels have the potential to provide a more functional and dynamic account of cellular behavior, proteomics has the potential to substantially increase understanding of the cancer phenotype (45). This may be particularly important for the many sarcoma subtypes that have few driver mutations but extensive copy number variation which is likely to result in complex alterations in protein expression levels (46–48). Multi-omic analyses show imperfect correlation of RNA and proteomic findings (49–52), which implies that proteomics may identify novel information that has not been revealed through genomic or transcriptomic research to date. An illustration of the consequences of this for individual patients is a case of chondrosarcoma where CDK4 gene amplification was identified by RNA sequencing and a CDK4/6 inhibitor was commenced according to the recommendation of a molecular tumor board (53). No response was seen, however, which appears to be explained by proteomic data showing that proteins involved in cell cycle regulation were not upregulated.

Although there have been major improvements in proteomic technology in recent decades, it has not progressed at the same rate as for genomics. Proteomic studies of cancer biology are at relatively early stage, and there are very few examples of its application in routine clinical oncology (54). Many proteomic studies in sarcoma have involved cohorts of small size or analysis with limited proteomic depth. Recent advances, however, have resulted in MS-based technologies which are able to detect a substantial proportion of the cancer proteome with relatively high sample throughput, including use of formalin-fixed paraffin-embedded (FFPE) cancer tissues collected as part of routine clinical care (55–57). These newer technologies enable the proteomes of fresh, frozen or FFPE tumor material to be analyzed in a short, clinically-applicable time frame on a large scale (57, 58), which could facilitate rapid translation of discoveries from cancer proteomics studies into clinical practice. In addition to proteome-wide studies, MS-based technologies also can be used for measurement of individual proteins and small panels of proteins relevant for clinical decision making, using techniques such as Multiple Reaction Monitoring (MRM) assays, which could be adopted into clinical practice.

The primary aim of this review is to describe key quantitative proteomic sarcoma studies with positive findings that pertain to clinical utility. First, we briefly define proteomic techniques that have been used in human sarcoma research, including recent advances in MS-based proteomic technology. We review studies that illustrate how proteomics may aid diagnosis and improve disease classification by distinguishing sarcoma histologies and identify distinct profiles within histological subtypes which may aid understanding of disease heterogeneity. We also review proteomic studies where proteomics has been applied to identify prognostic, predictive and therapeutic biomarkers, and specifically those that have utilized human tumor tissue and where findings have been clinically correlated. Cell line studies have been included where findings have been validated in clinically annotated human specimens or are accompanied by patient-derived xenografts. To conclude, we outline questions and unmet needs in sarcoma which can potentially be addressed with proteomics and propose future approaches to proteomic research.

## 2 Approaches to proteomics in sarcoma

Methods to identify and measure proteins can be divided into those that do or do not involve MS. Of the many available techniques, only those that have been used in human sarcoma research are described here.

### 2.1 Proteomic techniques not involving MS

Proteomic techniques not requiring MS that have been used in sarcoma research include gel electrophoresis and antibody-based techniques such as immunohistochemistry (IHC), western blotting, and protein microarrays. Antibody based proteomic research is limited by antibody-related issues, including reproducibility of antibody batches and specificity of epitope recognition in the protein of interest, requirement for prior knowledge of proteins of interest, and availability of validated antibodies (59).

Gel electrophoresis involves separation of proteins in a semisolid matrix based on mass and charge (60). Two-dimensional difference gel electrophoresis (2D DIGE) is the technique most frequently cited in sarcoma research. In this, protein samples are labelled with fluorescent dyes, mixed together, and separated on a gel across two dimensions. Dye-specific wavelengths are then detected to reveal different protein spots between samples. 2D DIGE is frequently combined with MS to identify proteins of interest. Here, proteins are separated using gel electrophoresis, selected protein spots are enzymatically digested into peptides, then analyzed with MS. Several thousand different proteins can be identified with gel electrophoresis, however gel-based techniques can be time-consuming and labor-intensive which presents a barrier to large-scale analysis. Underrepresentation of proteins is also a limitation (45).

Western blotting is a technique that allows identification of proteins of interest among a mixture of proteins (61). After proteins have been extracted from the tissue and separated in one dimension by gel electrophoresis, they are transferred (i.e., “blotted”) from the gel onto a membrane which is incubated with antibodies against the protein of interest. The bound antibodies are visualized and quantitated by methods such as immunostaining or immunofluorescence.

IHC, which is widely used in research and clinical practice, entails staining tissue sections on slides using antibodies to identify protein antigens, and a reagent coupled to the antibody that produces a stain (color reaction) to indicate the antigen’s location within the slide and its relative quantity *via* the staining intensity (62). IHC of FFPE tissue has been utilized in combination with quantitative proteomic techniques, or in integrative analyses, to validate findings.

Protein microarrays include reverse-phase protein arrays (RPPA) and antibody arrays. Either multiple tissue samples (RPPA), or a range of protein targeted antibodies (antibody arrays), are immobilized onto a microarray surface (63). These can then be incubated with an antibody or a tissue, respectively, to analyses hundreds of tissues samples or quantify proteins simultaneously. Analysis with protein arrays can thus be high-throughput, however study of more than few hundred proteins is generally not feasible.

### 2.2 MS-based proteomics

**Figure 1:**
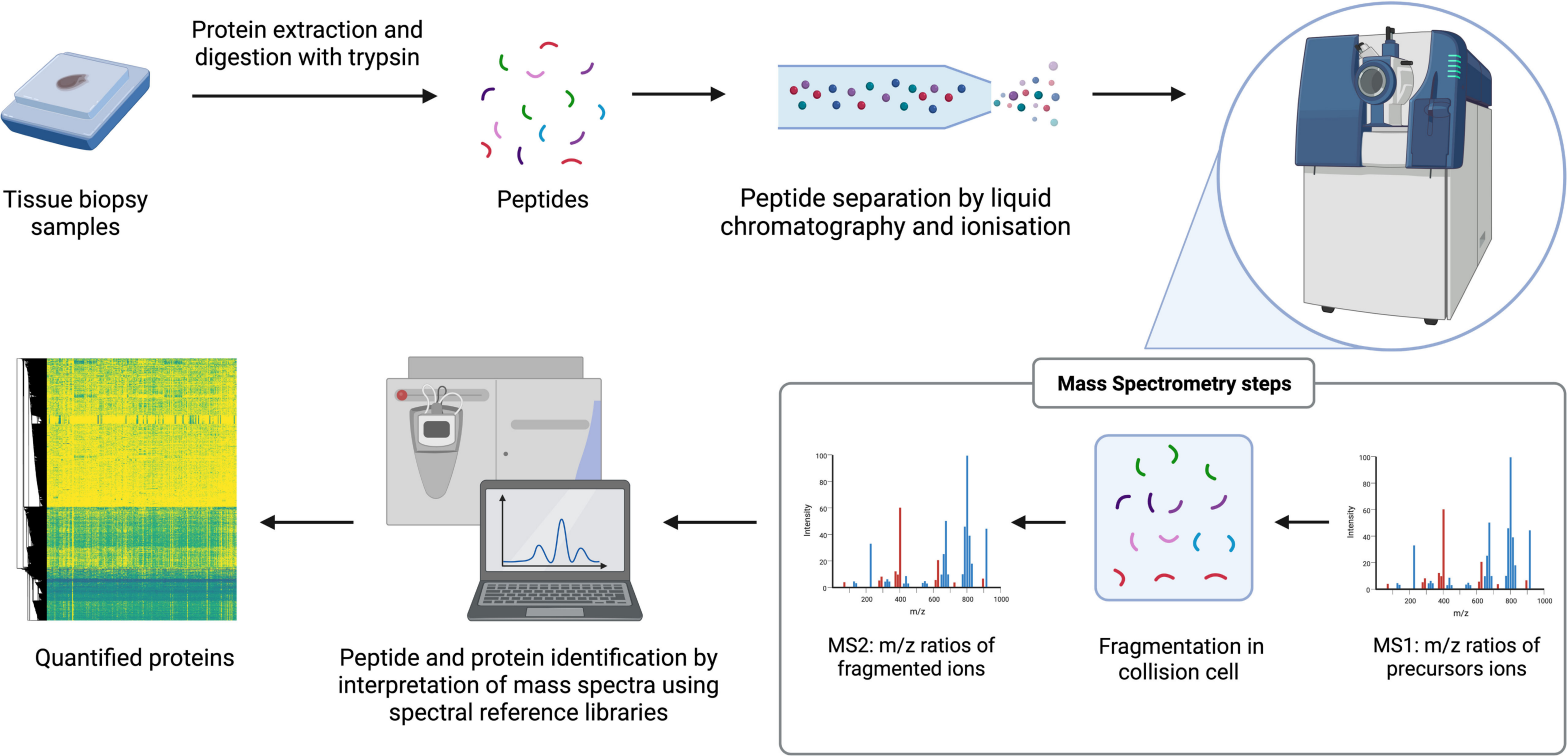
General workflow for label-free MS-based proteomics. The arrows reflect the stages of the workflow. e.g. starts with samples.

MS is a technique where proteins can be identified and quantified through analysis over time of the mass to charge (m/z) ratio of ionized peptides and of ionized fragments of those peptides (45, 55, 64). Tissue samples are prepared by lysis and enzymatic digestion of the released proteins to produce a peptide mixture that is fractionated over time by liquid chromatography (LC). Peptides from the LC gradient are injected into the mass spectrometers following ionization by techniques such as electrospray ionization (ESI) or matrix assisted laser desorption/ionization (MALDI). Within mass spectrometers, m/z ratios of precursor ions are first determined (MS1). Precursor ions are primarily the ionized digested peptides and are selected and fragmented in a collision cell then the m/z of the product ions generated from collision is determined (MS2). The product ions are ionized fragments of the peptides and provide partial amino acid sequence information. The combination of the precursor and product ion m/z is the key part of identifying the parent protein. Protein quantification can be achieved through label-free or isobaric labelling techniques. The latter approaches include tandem mass tagging (TMT) or isobaric tags for relative and absolute quantitation (iTRAQ). Label-free approaches include accurate monitoring of the intensity of detection of each peptide over LC elution time or can be approximated by counting the frequency in which the precursor ions are detected (spectral counting). Proteins are subsequently identified through analysis of peptide data with approaches including data dependent acquisition (DDA), which is often combined with isobaric labelling, or data independent acquisition (DIA). DIA techniques such as sequential window acquisition of all theoretical fragment ion spectra mass spectrometry (SWATH-MS), mostly require the generation of spectral reference libraries to interpret the mass spectrum generated without the need for chemical labels on the peptides. This procedure can detect thousands of proteins with relative quantitation from digested complex samples of multiple types (45, 55, 64). Advances in sample preparation techniques, instrumentation, and bioinformatics, have made it possible to conduct large-scale, reproducible, unbiased, proteomic discovery research (58). The main processes of a label-free MS-based proteomic workflow are illustrated in **Figure 1**.

## 3 Quantitative proteomic studies in sarcoma

Quantitative proteomic analyses in sarcoma are limited to date with less than 100 studies reported in the literature. Studies encompass broad analyses across multiple histological subtypes, and subtype-specific analyses. Sarcoma subtypes studied include GIST, osteosarcoma, chordoma, Ewing sarcoma, and various STS including RMS. There is a paucity of studies of the less common and ultra-rare sarcoma subtypes. Research has predominantly been conducted on frozen tissue samples and cell lines, with FFPE samples largely only used for IHC analyses until recently. Large quantitative proteomic analyses in sarcoma are limited in number with few dedicated proteomic studies; most large proteomic analyses feature as a component of multi-omic integrative molecular studies.

We have identified 23 key quantitative proteomic sarcoma studies with positive findings that pertain to clinical utility (**Table 1**). These studies encompassed histopathological subtypes of bone and STS, with potential clinical application for diagnosis, molecular subtyping, prognostication, and identification of novel therapeutic biomarkers and targets. Twenty of 23 studies incorporated MS-based techniques. The cohort sizes varied widely from less than 10 to almost 1000. Samples were from FFPE blocks (2 studies), frozen tissue (17 studies), cell lines (3 studies), or patient derived xenograft (1 study).

**Table 1:** Summary of quantitative proteomic sarcoma studies with potential clinical applications.

Study	Sarcoma subtype	Proteomic technique	Samples (n)	Sample type	Depth of coverage	Diagnostic biomarkers	Molecular subgroups within subtypes	Prognostic biomarkers	Therapeutic targets and biomarkers
Multi-subtype									
Goncalves (2022) (65)	*>40 all type cancer* *malignancies;* bone sarcoma, STS	MS	*949 including* 40, 22	Cell line	*8498 proteins* *-*				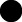
Zhang (2021) (66)	8 STS subtypes	RPPA	218	Frozen	220 proteins			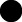	
Milighetti (2021) (67)	LMS, SS, UPS, DDLPS	MS	36	FFPE	2951 proteins	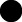	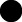	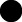	
TCGA (2017) (68)	LMS, DDLPS, UPS, MFS, SS, MPNST	RPPA	173	Frozen	192 proteins	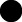	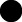		
Lou (2016) (69)	OS, MFS, UPS, LMS	MS	52	Frozen	20 diagnostic-, 9 survival- related proteins	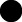		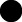	
Kirik (2014) (70)	STS mixed, LMS, UPS	2D DIGE MS	85, 38,16 0, 15, 5	Frozen Frozen	837 spots 778 proteins		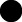		
Yang (2010) (71)	LMS, GIST	RPPA	31, 38	Frozen	7 proteins			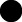	
Soft Tissue Sarcoma									
Toulmonde (2020) (72)	UPS	MS	25	FFPE	3512 proteins		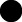		
Stewart (2018) (73)	RMS	MS -	12	O-PDX, myoblasts, myotubules	16,523 proteins 12,653 phosphosites		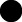		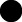
Bone Sarcoma									
Chaiyawat (2019) (74)	Osteosarcoma	2D DIGE MS	4 + 4 soft tissue callus	Frozen	329 spots 34 proteins				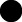
Cheng (2017) (75)	Osteosarcoma	MS	2 osteosarcoma, 1 osteoblast	Cell line	1549 proteins			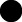	
Kubota (2013) (76)	Osteosarcoma	2D DIGE MS	13 -	Frozen	3494 spots 27 proteins				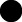
Kikuta (2010) (77)	Osteosarcoma	2D DIGE MS	12 -	Frozen	2250 spots 38 proteins				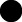
Hang (2022) (78)	Chordoma	MS	9	Frozen	5089 proteins 21,826 phosphosites				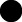
Shen (2021) (79)	Chordoma	MS	17	Frozen	4286 proteins			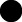	
Zhou (2010) (80)	Chordoma	2D DIGE MS	6 -	Frozen -	18 spots* 19 proteins			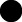	
Kikuta (2009) (81)	Ewing sarcoma	2D DIGE MS	8 -	Frozen -	2364 spots 66 proteins			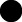	
Gastrointestinal Stromal Tumor									
Liu (2019) (82)	GIST	MS	13	Frozen	9177 proteins			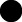	
Atay (2018) (83)	GIST	MS Western blot	12 17	Cell line Plasma	1606 proteins 30+ proteins				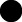
Ichikawa (2015) (84)	GIST	MS	8	Frozen	2550 proteins			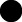	
Kikuta (2012) (85)	GIST	2D DIGE MS	17** -	Frozen -	3260 spots 25 proteins			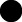	
Da Riva (2011) (86)	GIST	1D PAGE MS	16	Frozen	Not reported 39 proteins				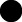
Suehara (2008) (87)	GIST	2D DIGE MS	17** -	Frozen	1513 spots 25 proteins			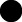	

*differentially expressed proteins between groups, total protein spots not reported.

**same samples of primary GIST tumors.

1D PAGE, 1D polyacrylamide gel electrophoresis; 2D DIGE, 2D directional gel electrophoresis; DDLPS, dedifferentiated liposarcoma; FFPE, Formalin fixed paraffin embedded tissue; GIST, gastrointestinal stromal tumor; LMS, leiomyosarcoma; MFS, myxofibrosarcoma; MPNST, malignant peripheral nerve sheath tumor; O-PDX, orthotopic patient-derived xenograft; OS, osteosarcoma; RMS, rhabdomyosarcoma; MS, mass spectrometry; RPPA, reverse phase protein array; SS, synovial sarcoma; STS, soft tissue sarcoma; UPS, undifferentiated pleomorphic sarcoma.

### 3.1 Distinguish sarcoma histological subtypes and aid diagnosis

Diagnosing sarcoma subtypes by histopathology is frequently difficult and many subtypes do not have definitive diagnostic markers (6, 7). Quantitative analyses of larger cohorts suggests that some sarcoma subtypes may possess unique proteomic profiles. This is of clinical interest given these signatures could be independently diagnostic, or enable differentiation from other sarcoma subtypes, and thus aid histological diagnosis.

Milighetti et al. (2021) examined 36 FFPE samples of localized disease of four sarcoma subtypes using SWATH-MS proteomics (67). Samples included 12 LMS, 10 UPS, 7 synovial sarcomas (SS) and 7 dedifferentiated liposarcoma (DDLPS) from various anatomical sites. The group quantified 2951 proteins across all cases and found 277 proteins to be significantly upregulated across the four histotypes. They demonstrated the sarcoma subtypes to have unique proteomic identities through three-dimensional t-Distributed Stochastic Neighbor Embedding (3D-tSNE) plot visualization where separation of the four distinct histological subtypes was seen. In hierarchical clustering, LMS and SS demonstrated distinct separation from other samples. Two groups, containing a mixture of UPS and DDLPS, also emerged. It is of relevance to note these defining signatures were maintained across anatomical sites. Differentially expressed proteins and networks were assessed for each subtype. For LMS and SS, 95 and 103 proteins respectively were significantly upregulated. In contrast to LMS and SS, in UPS and DDLPS, differential expression analysis revealed only 29 and 13 proteins respectively to be significantly upregulated. This study demonstrates sarcoma subtypes can possess unique proteomic profiles and thus the potential utility of proteomics for disease classification. It is of interest that some subtypes appear to possess more distinct proteomic profiles as reflected by higher numbers of differentially expressed proteins. This may mean that distinguishing some sarcoma subtypes through proteomic profiling may be more feasible than others. Alternatively, larger sample sizes may be required to identify defining profiles for more heterogeneous subtypes.

The finding of LMS and SS possessing distinct profiles was shared by The Cancer Genome Atlas (TCGA) consortium’s multi-omic integrative analysis (2017) which examined 173 frozen sarcoma specimens using RPPA-based proteomic analysis (68). Six histological subtypes were analyzed including 60 LMS, 46 DDLPS, 41 UPS, 15 myxofibrosarcoma (MFS), 6 SS and 5 malignant peripheral nerve sheath tumors (MPNST). Unsupervised consensus clustering of RPPA data with 192 antibodies identified five proteomic clusters with a statistically significant association with histologic type. Clustering of most LMS cases together was seen (cluster 1 included 48 of 53 LMS samples) with distribution of the other histological subtypes among the remaining groups. SS, which were limited in number (n=6) relative to other subtypes, did not form a distinct cluster, although all but one case (5 of 6 cases) appeared to fall into a single cluster C3 of mixed subtype. Pathway analysis of proteomic clusters identified an association of the LMS-enriched proteomic cluster with lower inferred activity of the apoptosis pathway, higher estrogen and progesterone receptor levels, and higher PI3K/AKT pathway activity relative to other clusters. In integrative analysis, both LMS and SS were found to be distinct from other sarcomas. LMS dominated a single multi-platform cluster (iCluster c1, 64 of 65 cases LMS) although SS was reported to be the most distinct sarcoma across all platforms with relatively uniform and unique patterns of DNA methylation, miRNA expression, and gene expression.

Lou et al. (2016) uniquely examined a cohort of bone and STS samples to evaluate if proteomics could identify diagnostic markers. Four proteins were identified which showed differential expression in LMS relative to MFS (69). MALDI-MS imaging (MALDI-MSI) was used to examine 52 fresh frozen LMS, UPS, MFS and high-grade osteosarcoma samples. Protein signals were considered diagnostic if they showed significant intensity differences specific to a single tumor type. Twenty protein signals were identified from well differentiated regions of LMS (n=4), MFS (n=9) and OS (n=9). Four proteins were identified from these protein signals through comparison with published reference library datasets: Acyl-CoA-binding protein, thioredoxin, macrophage migration inhibitory factor and galectin-1, all of which were highly expressed in LMS and showed low expression in MFS.

### 3.2 Identification of distinct profiles within histologic subtypes

Within sarcoma subtypes, there is heterogeneity in natural history and treatment response, suggesting there may be subgroups within histological subtypes. Identification of distinct proteomic profiles within histologic subtypes may help explain these differences, and in turn could assist in prognostication and treatment selection. Several proteomic studies have searched for subgroupings within individual subtypes that correlate with clinical outcomes, some of which have suggested subgroupings that cross subtype boundaries.

#### 3.2.1 Undifferentiated pleomorphic sarcoma and liposarcoma

UPS, as its name suggests, is a poorly differentiated tumor of unclear origin. It does not possess definitive diagnostic markers and subtype diagnosis is through exclusion of others. There is emerging proteomic evidence for molecular subgroups within UPS and potential shared biology with some liposarcomas. Subgrouping within UPS would be of considerable interest given its perceived heterogeneous nature and with emerging evidence for immunotherapy efficacy in UPS. It is estimated 20-30% of UPS respond to immunotherapy and it is currently not possible to predict which patients will respond (88, 89).

Toulmonde et al. (2020) undertook a comprehensive multi-omic analysis of UPS and demonstrated two main subgroups with distinct immunological and clinical features and therapeutic sensitivities (72). Their work incorporated protein analyses (MS, IHC and western blot), RNA and whole exome sequencing, and radiomics. The authors also generated cell lines and orthotopic patient derived xenograft (O-PDX) models from some of the tumors for functional studies. Proteomic analysis of 25 UPS samples with DIA LC-MS identified 3215 proteins. Unsupervised consensus and hierarchical clustering identified three groups (designated PA, PB, and PC) with 565 proteins differentially expressed between groups PA and PB (fold change ≥2, p 0.01). Gene set enrichment analysis revealed 19 pathways that were differentially expressed in the PA and PB groups. Upregulation of immune response pathways was noted in the PB group (consequently classified as immune high) and epithelial mesenchymal transition (EMT) and MYC target pathways in the PA group (immune low). Toulmonde at al. completed transcriptomic analysis of the same samples and unsupervised consensus and hierarchical clustering of this revealed three gene clusters (72). One group (group A) was enriched in genes involved in normal development, stemness and oncogenesis, and another (group B) in immune based genes. They found immune infiltrate IHC patterns (M2 macrophage density, and expression of IDO1, CD8 and PD-1) to be highly predictive of the gene expression classification and consequently termed the groups ‘immune low’ (group A), ‘immune high’ (group B) and ‘other’ (group C, n=4). They report high concordance between the proteomic and RNA clusterings (precision 82%) and good correlation of 114 related protein/gene pairs that were differentially expressed in both groups in each analysis (analysis of agreement, Spearman = 0.56, Pearson = 0.57, regression slope = 0.73). Additional findings included a 9-feature radiomic signature, through analyzing 14 pre-treatment MRIs, that could discriminate the ‘immune-high’ UPS from other UPS (specificity 100% (7/7), sensitivity 86% (6/7) accuracy of 93% (13/14)) and selective FGFR activity in immune low UPS, in cell lines and PDX models.

The concept of proteomic and immune-related molecular subgroupings is supported by Lazcano et al. (2022), who undertook an IHC immune marker study to assess the immune landscape of UPS (90). Through unsupervised clustering, the authors identified three immunologically distinct subgroups, termed ‘immune high, intermediate and low’ respectively, with different survival outcomes.

The hypothesis that some DDLPS share biology with a subgroup of UPS is supported by the Milighetti et al. MS-based study and the TCGA RPPA study. In the study by Milighetti et al. (2021) who performed hierarchical clustering of 36 FFPE samples of four sarcoma subtypes, UPS and DDLPS fell into two clusters, with clusters 2 and 4 both containing a mix of both subtypes (67). In the TCGA RPPA study (2017), which included 173 specimens of which 87 were DDLPS and UPS, although not commented upon by the authors, the majority of UPS cases fell into two RPPA clusters C2 and C5 (46). For DDLPS, approximately half of the cases appear to fall into shared RPPA cluster C5, which was comprised almost entirely of UPS and DDLPS, and the other DDLPS cases fell into other groups. In the integrated analysis, similar findings were observed where both subtypes fell into two clusters and included a shared cluster C3. UPS appear to cluster into two integrated clusters, C3 and C5, and DDLPS appear to fall into clusters C2 and C3.

These studies collectively suggest the hypothesis that despite the heterogeneous nature of UPS, most will fall into identifiable molecular subgroups, of which one may have an immune basis. Additionally, some UPS may have shared biological features with some DDLPS tumors.

#### 3.2.2 Rhabdomyosarcoma

RMS has traditionally been classified into several subtypes based on histopathological features. Subtypes include embryonal (ERMS), alveolar (ARMS), spindle cell and sclerosing RMS, and pleomorphic RMS (91). This classification has been questioned with discovery of genomic findings that appeared to better predict prognosis. ARMS were traditionally understood to follow a more aggressive trajectory and be associated with inferior prognosis compared to ERMS. Approximately 60% of ARMS have a PAX3-FOXO1 fusion gene and fusion-negative ARMS were subsequently shown to have a similar prognosis to ERMS. Consequently, a complex risk stratification system of clinicopathological and genomic variables associated with prognosis, including FOXO1 gene fusion status, has recently been adopted to guide treatment for RMS. There is, therefore, great potential for proteomics to deconvolute the interplay of histological and genomic findings.

Stewart et al. (2018) conducted a multi-omic analysis of RMS, which included quantitative proteomic analysis, and demonstrated differing proteomic profiles of ARMS, ERMS and RMS precursor cells (73). Their work also included genomic, epigenomic, and phosphoproteomic analyses with the aim of elucidating cellular origins and therapeutic vulnerabilities. Proteomic analysis with isobaric labelled TMT LC-MS was undertaken on myoblasts, myotubes and O-PDX RMS samples. PDX models were derived from recurrent RMS tumors and samples included 8 embryonal RMS (ERMS), and 4 alveolar RMS (ARMS). The authors identified 16,523 proteins and 12,653 phosphosites. Proteomic principal component analysis demonstrated separation of ERMS, ARMS, and myotubes and myoblasts. Differential expression analysis revealed differential expression of proteins MYOG and MYF5 between ERMS and ARMS. For 17 RMS tumors and their matched PDX there was agreement between IHC staining, gene expression and proteomic data for MYF5 and MYOG. Despite the small sample size, this study suggests that histopathological subgroups in RMS can be distinguished with proteomics and that exploring larger cohorts of tumors annotated with genomic fusion status and clinical outcomes using quantitative proteomic technology may increase our understanding of RMS and potentially assist in refining RMS classification.

#### 3.2.3 Leiomyosarcoma

LMS is one of the most common STS subtypes and one in which the underlying molecular biology has been studied more extensively than other sarcoma subtypes. The finding of two or more molecular subgroups in LMS has been suggested in several genomic, transcriptomic, and integrative studies (92–95). One proteomic study has suggested there may be three proteomic subgroups (70).

Kirik et al. (2014) analyzed 139 mixed subtype STS samples by 2D DIGE followed by TMT-based ESI-MS/MS analysis of 15 LMS and 5 myogenic UPS frozen tissue samples (70). The samples used for MS analysis were chosen on the basis of hierarchical clustering of the 2D DIGE results. Through MS analysis, 778 proteins were quantified and principal component analysis identified three LMS-enriched groups (LMS-A, -B and -C) and one UPS-enriched group. Three proteins (vinculin, COL6A3 and MYH11) were identified to be able to discriminate among the four groups. Unsupervised clustering showed group LMS-A appeared more distinct from tumors in groups LMS-B and LMS-C. Differential pathway analysis among the three LMS groups indicated apoptosis, cytoskeleton remodelling, and telomere regulation to be differentially regulated among these subgroups. An additional finding was an association of three ‘significantly deregulated’ proteins (vinculin, Integrin-linked protein kinase (ILK) and Creatinine Kinase type B) to be associated with vascular invasion, a negative prognostic factor in STS, although it was noted that a strong conclusion could not be drawn on account of the small sample size with only four cases with vascular invasion.

### 3.3 Prognostic proteomic biomarkers

Prognostic and predictive biomarkers have important clinical applications to identify patients with cancer at highest risk of relapse, to tailor treatment, and guide surveillance intensity. Several studies have endeavoured to identify prognostic proteomic biomarkers for sarcomas utilizing a range of proteomic techniques.

#### 3.3.1 Gastrointestinal stromal tumor

GIST is the sarcoma subtype that has been most extensively studied with respect to proteomic prognostic markers. GIST is the most common mesenchymal malignancy of the gastrointestinal (GI) tract (96). Primary disease predominantly arises in the stomach and small intestine, in 60-65% and 20-35% of cases respectively, although it may arise anywhere along the GI tract. The genomic landscape of GIST has been well characterized and GIST may be classified as KIT-mutated (67%), PDGFR-mutated (16%) or GIST without KIT or PDGFR mutations (16-17%) in which other alterations are recognized including NF1, SDH, BRAF or NTRK gene alterations. Molecular findings in GIST are known to correlate with variations in prognosis and therapeutic sensitivities. GIST with KIT exon 11 deletions on codons 557 and 558, for example, have much higher risk of relapse than others (97). Clinico-pathological features identified to be associated with distant recurrence of localized disease include tumor size, site, mitotic index and tumor rupture (96). Proteomic analyses, across GISTs of different sites and risk groups, propose several potential prognostic markers which include pfetin, ATP-dependent RNA helicase DDX39 (DDX39), promyelocytic leukaemia protein (PML), and protein tyrosine phosphatase nonreceptor type 1 (PTPN1) (82, 84, 85, 87).

Suehara et al. (2008) used 2D DIGE and MS to assess for prognostic biomarkers in 17 GIST which were classified into good- and poor-prognosis groups (87). Eight cases were classified as good prognosis (G-GIST) who had no evidence of metastases two years following surgery and ‘low to intermediate’ risk histopathology. Nine cases were defined as poor prognosis (P-GIST) who had metastases at diagnoses, or developed metastases within one year of resection, and ‘high risk’ histopathological findings. Forty-three protein spots, corresponding to 25 gene products, were found to differ between the two groups. Potassium channel protein pfetin was identified as a potential biomarker of favorable prognosis. Pfetin IHC was performed in a series of 210 cases and positive expression was found to be associated with superior 5-year metastasis-free survival (93.9% versus 36.2% for pfetin-positive and pfetin-negative cases respectively). Strong correlation between pfetin expression and established prognostic clinicopathological variables in GIST was seen in both uni- and multi-variate analyses. Pfetin was determined to be an independent prognostic risk factor among variables including risk classification and c-KIT and PDGFR mutational status. When high- and low-risk GIST groups were examined individually, pfetin-positivity conferred superior prognosis in both groups. The authors validated their prognostic findings with IHC in multiple independent cohorts (98, 99). Cumulatively, a total of 371 patients were assessed with IHC and, in meta-analysis, metastasis-free survival was 93.8% and 40.6% in pfetin-positive and -negative GIST, respectively.

Kikuta et al. (2012) examined differential protein expression of the same group of 17 GIST cases with a larger format gel electrophoresis device (85). Using 2D DIGE, 3260 protein spots were identified of which 38 protein spot intensities differed between P-GIST and G-GIST groups. These were analyzed with LC-MS and identified to correspond with 25 gene products. They contrasted their findings with those of their previous study and observed four shared proteins in both studies (pfetin, DDX39, superoxide dismutase and actin). DDX39 was selected for further evaluation with IHC in a cohort of 72 GIST patients. The function of DDX39 is unconfirmed although it has been reported to be a novel growth-associated RNA-helicase and to be required for global genome integrity and telomere maintenance. An association of inferior disease-free survival rates with strong DDX39 expression was observed (50.38% versus 90.2% for cases with DDX39-strong and DDX39-weak expression respectively). In a multi-variate analysis, which included clinical and pathological risk factors, DDX39 was determined to be an independent prognostic risk factor of disease-free survival. The prognostic value was then assessed in clinicopathological risk groups. A statistically significant difference in the survival was observed in low-risk group patients, with DDX39-strong or -weak expression, but this was not observed in intermediate- and high-risk patient groups.

Subsequent to these studies, MS has been used for GIST biomarker discovery. Ichikawa et al. (2015) conducted an integrated ESI-MS/MS proteomic and transcriptomic analysis in eight GIST samples and identified positive expression of the tumour suppressor PML protein to be associated with superior survival outcomes (84). The proteomic profiles of four gastric and four intestinal GISTs that had been surgically resected were compared. Differential expression analysis revealed 54 proteins to differ significantly between gastric and intestinal sites. Low expression of PML was identified in intestinal GIST and validated through PML IHC evaluation in a cohort 254 GIST cases. An association of positive PML IHC expression with superior 5-year recurrence free survival rates was reported (91.7% versus 60.1% for PML-positive and -negative cases respectively).

Liu et al. (2019) have more recently used TMT-based MS (LC-MS/MS) to comprehensively examine the proteomic profile of GIST tissue and identified phosphatase PTPN1 as a potential prognostic biomarker (82). Thirteen patients of different National Institute of Health (NIH) risk groups were analyzed. The NIH consensus classification stratifies GIST patients into four risk subgroups based on tumor size and mitotic rate. The group quantified 9177 proteins of which 4930 proteins were observed in all GIST cases. 187 proteins were observed to be downregulated and 517 proteins to be upregulated, including phosphatase PTPN1. To evaluate the reliability of their proteomic approach, comparison among risk groups of proteins in their dataset was made with well-known over-expressed proteins in GIST. Changing patterns of protein expression between groups were observed and consistent with previous reports. As a previously undescribed finding in GIST, although understood to be associated with metastases in other malignancies, PTPN1 expression and its correlation with survival outcomes was assessed with IHC in an extended cohort of 117 clinically annotated cases. Higher PTPN1 expression was observed in NIH lower risk cases, relative to higher risk cases (p=0.037) and associated with numerically lower rates of metastatic disease. The proportions of metastatic cases in PTPN1-high, -intermediate and -low expression were 0/25, 3/49 and 4/43 respectively.

#### 3.3.2 Leiomyosarcoma

In LMS, RPPA-based analysis has been used to identify prognostic protein biomarkers. In an early study by Yang et al. (2010), expression of an epithelial marker E-cadherin has been observed to be associated with improved survival (71). In combination with transcriptomic analysis, 31 LMS and 38 GIST samples were analyzed by RPPA. Protein expression of E-cadherin was not seen in GIST although it was identified in a subset of LMS cases. Expression of additional epithelial markers in LMS were also observed (epithelial membrane antigen, cytokeratin AE1/AE3 and pan cytokeratin). These findings were confirmed with western blotting, and IHC, and e-cadherin positive LMS (n=18) were observed to have better total survival than LMS with no e-cadherin expression (n=13). Transcriptomic analysis, of the same group of patients, mirrored this finding with higher survival rates noted in patients with elevated mRNA levels of epithelial markers than patients with higher mRNA levels of mesenchymal cell marker genes.

#### 3.3.3 Chordoma

In bone sarcoma chordoma, Zhou et al. (2010) examined frozen samples of primary disease tissue of six patients, using 2D DIGE and MALDI-MS (80). Fourteen upregulated proteins and five downregulated proteins were identified and proteins alpha enolase (ENO1), pyruvate kinase M2 (PKM2) and gp96 were chosen for further analysis. In paired tissue analyses, their expression was noted to be greater in chordoma than in adjacent normal tissues, and higher in recurrent cases than primary tumors. The association of survival endpoints with IHC expression of these proteins was subsequently assessed in a cohort of 37 patients. ENO1 and PKM2 were associated with disease-free survival in univariate analysis however no proteins were found to be prognostic in multi-variate analysis of disease-free survival nor univariate analysis of overall survival.

In a later study, Shen et al. (2021) utilized LC-MS/MS to identify the enzyme asparagine synthetase (ASNS) as a novel prognostic biomarker of recurrence in chordoma of the skull base (79). Frozen samples of 17 patients were analyzed and the proteomic findings of nine cases who had short recurrence free survival (RFS) intervals were compared with eight cases with long RFS intervals. In total, 4286 proteins were quantified across all patients with 146 and 112 proteins identified to be up- and down- regulated respectively in the rapid-recurrence group. Pathway analysis of upregulated proteins suggested alanine, aspartate and glutamate metabolism was a main pathway in the rapid recurrence groups. ASNS, an enzyme which catalyses the conversion of aspartic acid to asparagine, was selected for prognostic evaluation based on literature review. In two cohorts of 93 and 94 patients respectively, an association of shorter RFS with high IHC expression of ASNS was found. In the training cohort of 93 patients, a median RFS of 24.5 months was observed in ASNS-high cases relative to 59.0 months in ASNS-low cases (p=0.009) and ASNS was found to be an independent prognostic factor in univariate and multivariate Cox regression analysis. In a functional experiment to evaluate the impact ASNS in oncogenesis, knockdown of ASNS in chordoma cells (UM-Chorl and MUG-Chor1) by siRNA resulted in inhibition of cell growth, colony formation, migration and invasion.

#### 3.3.4 Osteosarcoma

Cheng et al. (2017) identified proteins minichromosome maintenance protein 2 (MCM2) and minichromosome maintenance protein 3 (MCM3) through MS analysis of osteosarcoma cell lines and demonstrated an association of positive IHC expression with inferior tumor free- and overall- survival in 129 tissue samples (75). The group performed SWATH-MS analysis of one osteoblast and two osteosarcoma cells lines to identify proteins associated with carcinogenesis. Of 1549 proteins quantified, 62 upregulated proteins and 87 downregulated proteins were identified in all osteosarcoma cell lines compared to the osteoblast cell line. Functional screening was conducted on nine chosen proteins and inhibition of proteins MCM2 and MCM3 were found to reduce osteosarcoma cell proliferation. Knockdown of MCM2 and MCM3 significantly inhibited osteosarcoma growth *in vitro* and *in vitro*. To determine the clinico-pathological significance of these two proteins, IHC expression of MCM2 and MCM3 was assessed in 129 clinically annotated FFPE tissue samples. Higher expression of MCM2 and MCM3 was found to correlate with disease recurrence and both proteins were found to be independent prognostic risk factors for tumor free- and overall- survival.

#### 3.3.5 Ewing sarcoma

Kikuta et al. (2009) used 2D DIGE and MS to search for prognostic biomarkers in the biopsy samples of eight Ewing sarcoma patients (81). Five samples were classified as poor-prognosis (deceased with disease within two years of diagnosis) and three as good prognosis (alive with no evidence of disease over three years from diagnosis). Using 2D DIGE, 2364 protein spots were identified. Sixty-six protein spots, corresponding to 53 gene products including the protein nucleophosmin, were found to differ between good- and poor- prognosis samples. Nucleophosmin was chosen for further evaluation because it has been associated with carcinogenesis and tumor progression in other malignancies. IHC validation and prognostic assessment was undertaken in an independent cohort of 34 clinically annotated cases. Nucleophosmin positivity was observed in 23 of 34 cases and associated with shorter overall survival in uni- and multi-variate analyses.

#### 3.3.6 Multi-subtype prognostic analyses

Lou et al. (2016) utilized MALDI-MS imaging (MALDI-MSI), as described above in section 3.1, to determine if proteins could differentiate clinically important outcomes including survival and development of metastases (69). Protein signals associated with overall survival were investigated within individual subtypes (LMS, UPS, MFS and high-grade osteosarcoma) and a combined soft tissue cohort which included the LMS, UPS and MFS cases. Nine protein signals were identified. Assignable proteins (n=4), where high intensity was associated with poor survival, included proteasome activator complex subunit 1 (PSME1, in STS patients), two histone H4 variants (in LMS patients) and haemoglobin subunit beta (in MFS patients). All of these proteins have previously been linked to cancer progression and pathogenesis, including being involved in microvascular invasion and stromal activation. One unclassified protein signal (m/z 8093) displayed a possible association with metastasis-free survival in UPS patients (p-value of 0.03 in Kaplan–Meier analysis, p-value of 0.06 in multivariate analysis). IHC expression was performed on FFPE samples from the same patients and validated that higher expression of PSME1 was associated with poor prognosis. A novel component of this analysis considered if intra-tumoral heterogeneity may also impact prognosis. Samples were histopathologically annotated to ascertain well-, moderately- and un-differentiated regions. MALDI-MSI data of these regions within samples were subsequently interrogated. This resulted in identification of two additional proteins (cytochrome C oxidase subunit 2 and unclassified protein signal m/z 6281). When prognostic biomarker assessment was limited only to undifferentiated regions, these proteins corresponded with poor prognosis in LMS. They also assessed for proteomic subpopulations within the differentiated regions. Through virtual microdissection, two molecularly distinct subpopulations were identified within moderately differentiated regions of osteosarcoma samples, that were statistically associated with different overall survival.

More recently, Milighetti et al. (2021) used MS-based proteomics, as described above in section 3.1, to identify a multi-protein expression profile associated with shorter overall survival in a subgroup of STS cases (67). Hierarchical clustering of 36 cases based on the expression values of 133 proteins, selected with p <0.05 in univariable Cox regression analysis, revealed three subgroups of mixed histological subtypes associated with significantly different overall survival. Group 2 showed an inferior outcome and was comprised of 3 DDLPS, 3 LMS and one UPS. The proteomic subgroups remained an independent prognostic factor after adjusting for other clinicopathological factors including age, tumor size, grade, sex and histological subtypes in multivariable Cox regression analysis.

A multi-subtype multi-protein prognostic risk signature has also been generated from RPPA-based protein data. The Cancer Proteome Atlas (TCPA) is an open-access resource of RRPA-based proteomics data of many tumor subtypes (100). The TCPA have analyzed an RPPA of 221 sarcomas comprising 8 different soft tissue subtypes with 220 antibodies. This public dataset has recently been interrogated by Zhang et al. (2021) to reveal 55 proteins, from 218 samples, to be associated with overall survival (66). Individual patient risk scores were calculated, based on corresponding protein expression data multiplied by the Cox regression coefficient. Dichotomised risk groups (high or low risk) were then created based on median risk score. A risk signature consisting of 7 proteins was independently associated with overall survival after adjusting for clinicopathological parameters. Of note, the prognostic utility of the signature in predicting overall survival within some clinicopathologic subgroups (e.g., for LMS, UPS, both younger and older age, both positive and negative margin status) but not others (DDLPS, MFS). The signature was also associated with a worse progression free interval (PFI) (p <0.0001), and a trend towards a shorter disease free interval (DFI) (p=0.09) in high-risk patients.

The findings of many of these studies require validation with independent cohorts and larger sample sizes for clinical application. The demonstration of individual proteins and proteomic subgroups being associated with survival, however, is supportive of the utility of proteomics for prognostic biomarker discovery. It is intriguing to speculate that it may be possible to identify a subtype-agnostic proteomic survival signature, as illustrated by Milighetti et al. MS-based work and the study of TCPA RPPA data. (66, 67).

### 3.4 Proteomic therapeutic targets and biomarkers

Given the established utility of proteins in oncology as diagnostic markers, drug targets, and as tumor markers for disease and therapeutic activity monitoring, it is anticipated that proteomics could reveal novel therapeutic biomarkers and targets in sarcoma.

#### 3.4.1 Therapeutic targets

Hang et al. (2022) undertook proteomic and phosphoproteomic analyses to identify pathway alterations and aberrant kinase activity in chordoma (78). They demonstrated suppression of chordoma cell growth in functional experiments of identified proteins. Four-dimensional label-free LC-MS/MS analysis of frozen chordoma tissue and paired normal muscle tissue from nine patients quantified 5089 proteins and 21,826 phosphosites from 4724 phosphorylated proteins. Pathway analysis of proteins differentially expressed in chordoma tissue revealed strong enrichment of oxidative phosphorylation. It is thought that chordomas arise from the embryonic notochord and that the Wnt-signalling pathway plays a crucial role in embryonic and notochord development. Ten of 100 known Wnt-signalling proteins were observed to be differentially expressed including a two-fold elevation of expression of β-catenin. Using an integrated computational approach that enables prediction of kinase activities based on differences in their substrate phosphorylation, 28 kinases with altered activity were found. Significant differences between chordoma and normal tissue were identified for three kinases: Aurora kinase A (AURA), Cyclin-dependent kinase 9 (CDK9) and MAPK/MAK/MRK overlapping kinase (MOK). Elevated expression of these kinases was verified using western blotting of proteins from chordoma and normal tissue of six chordoma patients independent from the patients examined by MS. All three proteins were expressed in tumor tissue, although some inconsistencies of AURA and CDK9 were noted; expression was observed in normal tissue of some cases and variable expression was seen in tumor samples. Knockdown of AURA, MOK and CDK9 in chordoma cell lines and treatment with CDK9 inhibitor AZD4573 were shown to compromise cell proliferation in functional experiments. The authors thus concluded these cell-proliferation related proteins may promote chordoma oncogenesis and may represent new therapeutic targets.

Stewart et al. (2018) used a multi-omic approach, described above in section 4.2.2, to identify dysregulated pathways in RMS and target these in preclinical models (73). To do this, they focused on upregulated pathways elucidated in integrated analysis that also demonstrated drug sensitivity in primary cultures of O-PDX models. Analysis of 1,767 drug/tumor combinations in the PDX models identified significant *in vitro* activity of a WEE1 kinase inhibitor, AZD1775, which targets the unfolded protein response and G2/M checkpoint pathways. Epigenetic, gene expression and proteomic/phosphoproteomic data for RMS relative to myoblasts revealed upregulation of this pathway. Application of AZD1775 to cell lines caused them to arrest in the G2/M phase of the cell cycle. When combined with irinotecan or vincristine, these drugs caused high levels of DNA damage. Pre-clinical placebo-controlled trials of AZD1775, as a monotherapy and in combination with irinotecan and vincristine, were then conducted in several O-PDX models. Overall survival was not improved with AZD1775 monotherapy compared to placebo although a compiled complete and partial response of 70% was seen with triplet combination therapy (AZD1775, irinotecan and vincristine). The authors conclude these findings suggest WEE1 may be a therapeutic target in RMS, thereby illustrating how proteomics may enable therapeutic discovery.

The utility of high-throughput quantitative proteomics for therapeutic discovery is supported a pan-cancer analysis of 949 human cell lines by our group (2022), which included 62 bone and soft tissue sarcoma cell lines (65). DIA MS-based analysis (SWATH-MS) of more than 40 histological cancer types, across 28 tissue types, quantified 8498 proteins. Integration of multi-omics, drug response, and CRISPR-Cas9 gene essentiality screening revealed thousands of protein biomarkers of cancer vulnerabilities. In bone sarcoma cell lines, drug/protein associations included a strong association of protein abundance of peptidyl-prolyl cis-trans isomerase H (PPIH) with response to the Aurora kinase B/C selective inhibitor GSK1070916. The study revealed thousands of protein biomarkers of cancer vulnerabilities, including the example detailed, that were not significant at the transcript level. Furthermore, the study also demonstrated that the predictive power of the proteome to predict drug response was overall very similar to that of the transcriptome.

The use of proteomics for therapeutic target discovery is in its infancy, however these studies support its potential utility for therapeutic discovery, and identification of novel therapeutic approaches.

#### 3.4.2 Predictive biomarkers

Identification of predictive biomarkers of disease outcomes including treatment response could better inform treatment decisions and improve survival of patients with sarcoma. Several studies, which are described below, have used cell lines and small cohorts of frozen tissues (5-30 samples) to identify individual proteins as biomarkers of treatment response.

In osteosarcoma, expression of peroxiredoxin 2 (PRDX2) and 78 kDa glucose-related protein (GRP78) have been reported to be associated with poor treatment response (74, 76, 77). One group performed two 2D DIGE studies to assess for proteomic biomarkers of osteosarcoma response to neo-adjuvant chemotherapy (76, 77). In both studies, which included 12 and 13 patients respectively, an association of increased peroxiredoxin 2 (PRDX2) expression with poor response to chemotherapy was reported. Frozen samples which had been obtained prior to treatment were analyzed and the proteomic findings of patients classified as good and poor responders were compared. Patients were classified based on the histological necrosis of their post-chemotherapy resection specimens where good response was defined as histological necrosis of greater than 90%. In the first study by Kikuta et al. (2010), 55 of 2250 identified protein spots, corresponding to 38 unique proteins, differed significantly between good and poor responders (77). In the second study by Kubota et al. (2013), 33 of 3494 protein spots, corresponding to 27 proteins, differed significantly between the two groups. MS was used to identify the differential spots and PRDX2 was noted to be upregulated in poor-responders in both studies. The chemotherapy regimens in each study were different, therefore it was noted to be of interest that upregulation of PRDX2 was identified in both studies. To investigate the association of PRDX2 with response to chemotherapy, Kubota et al. conducted experiments of siRNA-induced silencing of PRDX2 in three osteosarcoma cell lines (76). PRDX2 silencing resulted in increased sensitivity of osteosarcoma cells to chemotherapy (methotrexate, doxorubicin and cisplatin) and a decrease in cell proliferation, invasion and migration in functional assays. The authors propose PRDX2 may therefore represent a predictive biomarker and an indicator of chemotherapy resistance.

Chaiyawat et al. (2019) used 2D DIGE and LC-MS/MS to identify GRP78 to be differentially expressed in osteosarcoma when four frozen tissue osteosarcoma samples and four normal soft tissue callus samples were compared (74). In a separate validation cohort, an association of upregulation of GRP78 with poor treatment response was reported.

In GIST, positive expression of Stem Cell Growth Factor (SCGF) has been associated with response to the tyrosine kinase inhibitor imatinib (86), and exosomal expression of proteins Sprouty homolog 4 (SPRY4) and tyrosine protein kinase KIT have been associated with increased risk of tumor progression (83). Da Riva et al. (2011) examined resection samples of 16 GIST patients including 15 tumor samples that had been exposed to neo-adjuvant imatinib treatment and one treatment naïve sample (86). Cases were classified according to histological response in terms of percentage of viable cells and defined as high-, moderate- and non-responders. Gel electrophoresis, western blotting and MALDI-MS analysis of five samples, including three high-responders, identified haemopoietic growth factor SCGF expression in imatinib-responsive GIST tumors and its absence in some non-responding tumors. It was noted results were not consistent across all cases. IHC analysis of five cases showed SCGF to be restricted to the stromal compartment. Inflammatory infiltrates were detected in some imatinib-affected areas and the authors hypothesized an inflammatory reaction induced by imatinib treatment might be responsible for the SCGF positivity. Experiments revealed SCGF positivity in GIST marker KIT (CD117)-negative and CD68 (a marker of monocytic cells)-positive areas. The authors proposed that identification of SCGF may be consistent with an imatinib-induced inflammatory response but acknowledged further studies are necessary to determine the reason for its expression.

Atay et al. (2018) reported GIST-derived exosomal SPRY4 and KIT expression as biomarkers of disease status and imatinib response (83). LC-MS/MS was used to analyze GIST-derived exosomes from two GIST cell lines, GIST-T1 and GIST882. Exosomal proteins were quantified (n=1060) and a selection of proteins were validated using cell lines, patient-derived KIT+ exosomes and GIST tissues. Enrichment of multiple established GIST-associated protein markers was observed, including KIT, CD34, anoctamin-1 (ANO1), hypoxia inducible factor 1 alpha (HIF1α), and succinate dehydrogenase B (SDHB). Enrichment of protein SPRY4 was also observed which the group had previously identified as a genetic marker that could predict imatinib response (101). To explore the therapeutic value of proteins identified, immunoblot expression of SPRY4 and KIT was compared in paired pre- and post- imatinib plasma samples, with findings of reduced levels of SPRY4 and KIT post-imatinib in patients classified to be imatinib-responsive and higher levels in patients classified to be imatinib-resistant (83). Quantitative analysis of circulating exomes of the plasma of ten primary GIST and seven metastatic GIST revealed significantly increased levels of KIT and SPRY4 in metastatic patients. In imatinib-responsive primary GIST patients, both KIT and SPRY4 were significantly reduced. In imatinib-treated metastatic patients, only KIT was significantly elevated. To further verify their findings, IHC was performed in GIST patient tissue with a finding of significant expression of SPRY4 in metastatic GIST cases (n=7) compared with resected primary GIST cases (n=8). The authors conclude that expression of SPRY4 and KIT may represent markers of tumor progression and could be predictive of imatinib therapeutic response.

## 4 Outstanding questions and future approaches

The studies outlined here highlight the potential utility of quantitative proteomics for the study and clinical management of sarcomas. Detailed proteomic analyses are currently limited in scale and the proteomes of many sarcoma subtypes remain largely unexplored. The extensive clinicopathological heterogeneity among the large number of sarcoma subtypes makes the lack of large-scale studies especially problematic.

There are many questions and unmet needs in sarcoma which can potentially be addressed with proteomics. These are summarized in **Table 2** and encompass biomarker discovery and improving diagnosis, disease classification, risk stratification, and treatment. The scope for future research with the emerging proteomic technologies is therefore very large.

**Table 2:** Unmet needs and questions in sarcoma that may be possible to address with proteomics.

**Diagnosis**	• Identification of diagnostic biomarkers for subtypes that are challenging to classify or lack simple diagnostic markers e.g. UPS, LMS. • Development of diagnostic tools requiring tissue samples of smaller size thereby minimizing the invasive nature of biopsy for patients. *This is with consideration that in some cases core biopsies are insufficient to confirm diagnosis in sarcoma or genomic testing cannot be completed due to sample quality or available material.* • Development of alternatives to biopsy such as blood-based testing. • Diagnostic tools of improved practicality, e.g. currently evaluation of multiple IHC stains, with or without FISH or gene panels, along with histopathological examination are utilized to confirm diagnosis.
**Disease classification**	Establishing • Whether molecular subtypes should be considered separate entities or grouped together, e.g., round cell subtypes including CIC-rearranged and BCOR-altered sarcoma. • Whether histological subtype-agnostic molecular subtypes or classifications can be found which may better inform diagnosis, prognosis, and treatment.
**Risk stratification**	• Identification of prognostic biomarkers of localized and advanced disease. • Development of a more robust risk classification systems for localized disease to better inform treatment decisions that could be applied to individual or multiple sarcoma subtypes. either single biomarkers or multi-feature composite models combining clinicopathological and molecular features.
**Disease monitoring**	• Identification of tumor markers in sarcoma for disease activity monitoring. • Tumor markers could be of utility for surveillance of localized disease or as markers of therapeutic response for patients receiving treatment for advanced disease.
**Treatment**	• Identification of new drug targets and therapeutic strategies given treatment options in sarcoma are limited. • Improving the drug target yield of molecular findings. Most genomic findings currently are not actionable and downstream pathways of drivers remain to be elucidated. • Identify markers of resistance to established therapeutic options in sarcoma.
**Therapeutic Biology**	Improvement in biological understanding of • The basis of chemo-sensitivity seen within sarcoma subtypes, e.g., Ewing sarcoma, osteosarcoma, RMS, myxoid liposarcoma, UPS, cutaneous angiosarcoma. • What underlies chemotherapy resistance seen within sarcoma subtypes, e.g., alveolar soft part sarcoma, clear cell sarcoma, chondrosarcoma. • Biology and biomarkers of subtypes responsive to immunotherapy, e.g. ASPS, UPS, cutaneous angiosarcoma.
**Predictive Biomarkers**	Biomarkers of • Adjuvant chemotherapy efficacy (prognostic and predictive biomarkers). • Advanced disease treatment therapeutic response to inform treatment choice and sequencing. Identify those who preferentially respond, and underlying mechanisms, to standard treatment options, e.g., in STS: doxorubicin, gemcitabine/docetaxel, and pazopanib.

Multi-subtype studies and subtype-specific analyses are both needed. These will enable exploration and definition of the proteomic profiles of multiple different histological subtypes and allow investigation of similarities and differences across the spectrum of sarcoma. New molecularly-defined subtypes that may provide an improved basis for classifying and treating sarcoma could potentially emerge. Dedicated subtype-specific analyses would be of value to study the proteome of unexplored less common and ultra-rare sarcoma subtypes. For all subtypes, analysis of large cohorts would facilitate investigation of underlying biology, disease mechanisms, and molecular subgroups within sarcoma subtypes. Clinical annotation would help unravel the potential relevance of molecular subgroups. Discovery of diagnostic, prognostic and therapeutic biomarkers, would be of profound interest and could be explored in both subtype-specific analyses and multi-subtype studies.

Understanding disease biology will be the first step in transformative proteomic research. For maximum clinical impact, studies would consist of clinically annotated cohorts of large size, designed around clinical questions addressing unmet needs in sarcoma and including integrative multi-omic analysis incorporating both genomics and proteomics. Incorporation of phosphoproteomics and kinomics (also called chemoproteomics) may also be important, particularly for discovery of new therapeutic targets, because post translation modifications such as phosphorylation may have a major impact on protein function and many protein kinases are readily druggable (102–104). Studying disease at multiple time points from pre-malignant lesions to diagnosis to advanced disease, will help elucidate mechanisms of carcinogenesis, disease relapse, progression, and treatment resistance.

Challenges to conducting future research will be accruing sarcoma cohorts that are clinically annotated and of sufficient size, with timely validation of findings in independent cohorts. For translation of predictive and therapeutic biomarkers into clinical practice, validation in large, prospective clinical trials will likely be required. Proteomic challenges include technological limitations, such as identification of low abundant proteins, and cross platform comparability and validation. If novel discoveries are made, there will undoubtedly be clinical implementation challenges. A recent review has discussed DIA-MS challenges for cancer in more depth including barriers to clinical implementation and strategies to overcome these (54). In particular, this review highlights the critical importance of involving cancer clinicians in study design, and of international collaboration and data sharing.

## 5 Conclusions

We have reviewed recent quantitative proteomic findings and identified the potential for robust proteomic studies to address unmet clinical needs in sarcoma. It is anticipated that large scale, clinically annotated, integrated multi-omic analyses will facilitate novel discoveries pertaining to sarcoma diagnosis, risk stratification, and treatment, with the hope of improving outcomes and treatment options for sarcoma patients.

## Author contributions

EC wrote the manuscript. PR, RR, PG, and LH reviewed and edited the manuscript. All authors contributed to the article and approved the submitted version.

## References

[1] WHO Classification of Tumours Editorial Board. Soft tissue and bone tumours. In: WHO classification of tumours series, 5th ed, vol. 3. . Lyon (France: International Agency for Research on Cancer (2020). Available at: https://tumourclassification.iarc.who.int/chapters/33.

[2] Farid M Ngeow J. Sarcomas associated with genetic cancer predisposition syndromes: a review. Oncologist. (2016) 21(8):1002–13. doi: 10.1634/theoncologist.2016-0079 PMC4978564 27401891

[3] Howlader NNA Krapcho M Miller D Brest A Yu M Ruhl J Tatalovich Z Mariotto A Lewis DR Chen HS Feuer EJ Cronin KA eds. SEER cancer statistics review 1975-2018. Bethesda, MD: National Cancer Institute (2021). Available at: https://seer.cancer.gov/csr/1975_2018/.

[4] Stacchiotti S Frezza AM Blay JY Baldini EH Bonvalot S Bovee J. Ultra-rare sarcomas: a consensus paper from the connective tissue oncology society community of experts on the incidence threshold and the list of entities. Cancer. (2021) 127(16):2934–42. doi: 10.1002/cncr.33618 PMC8319065 33910263

[5] Schaefer IM Cote GM Hornick JL. Contemporary sarcoma diagnosis, genetics, and genomics. J Clin Oncol (2018) 36(2):101–10. doi: 10.1200/JCO.2017.74.9374 29220288

[6] Ray-Coquard I Montesco MC Coindre JM Dei Tos AP Lurkin A Ranchere-Vince D. Sarcoma: concordance between initial diagnosis and centralized expert review in a population-based study within three European regions. Ann Oncol (2012) 23(9):2442–9. doi: 10.1093/annonc/mdr610 PMC3425368 22331640

[7] Arbiser ZK Folpe AL Weiss SW. Consultative (expert) second opinions in soft tissue pathology. analysis of problem-prone diagnostic situations. Am J Clin Pathol (2001) 116(4):473–6. doi: 10.1309/425H-NW4W-XC9A-005H 11601130

[8] Callegaro D Miceli R Bonvalot S Ferguson P Strauss DC Levy A. Development and external validation of two nomograms to predict overall survival and occurrence of distant metastases in adults after surgical resection of localised soft-tissue sarcomas of the extremities: a retrospective analysis. Lancet Oncol (2016) 17(5):671–80. doi: 10.1016/S1470-2045(16)00010-3 27068860

[9] Bramer JA van Linge JH Grimer RJ Scholten RJ. Prognostic factors in localized extremity osteosarcoma: a systematic review. Eur J Surg Oncol (2009) 35(10):1030–6. doi: 10.1016/j.ejso.2009.01.011 19232880

[10] Marina NM Smeland S Bielack SS Bernstein M Jovic G Krailo MD. Comparison of MAPIE versus MAP in patients with a poor response to preoperative chemotherapy for newly diagnosed high-grade osteosarcoma (EURAMOS-1): an open-label, international, randomised controlled trial. Lancet Oncol (2016) 17(10):1396–408. doi: 10.1016/S1470-2045(16)30214-5 PMC5052459 27569442

[11] Woll PJ Reichardt P Le Cesne A Bonvalot S Azzarelli A Hoekstra HJ. Adjuvant chemotherapy with doxorubicin, ifosfamide, and lenograstim for resected soft-tissue sarcoma (EORTC 62931): a multicentre randomised controlled trial. Lancet Oncol (2012) 13(10):1045–54. doi: 10.1016/S1470-2045(12)70346-7 22954508

[12] Gronchi A Palmerini E Quagliuolo V Martin Broto J Lopez Pousa A Grignani G. Neoadjuvant chemotherapy in high-risk soft tissue sarcomas: final results of a randomized trial from Italian (ISG), Spanish (GEIS), French (FSG), and polish (PSG) sarcoma groups. J Clin Oncol (2020) 38(19):2178–86. doi: 10.1200/JCO.19.03289 32421444

[13] Gronchi A Stacchiotti S Verderio P Ferrari S Martin Broto J Lopez-Pousa A. Short, full-dose adjuvant chemotherapy (CT) in high-risk adult soft tissue sarcomas (STS): long-term follow-up of a randomized clinical trial from the Italian sarcoma group and the Spanish sarcoma group. Ann Oncol (2016) 27(12):2283–8. doi: 10.1093/annonc/mdw430 27733375

[14] Seddon B Strauss SJ Whelan J Leahy M Woll PJ Cowie F. Gemcitabine and docetaxel versus doxorubicin as first-line treatment in previously untreated advanced unresectable or metastatic soft-tissue sarcomas (GeDDiS): a randomised controlled phase 3 trial. Lancet Oncol (2017) 18(10):1397–410. doi: 10.1016/S1470-2045(17)30622-8 PMC5622179 28882536

[15] Tap WD Wagner AJ Schoffski P Martin-Broto J Krarup-Hansen A Ganjoo KN. Effect of doxorubicin plus olaratumab vs doxorubicin plus placebo on survival in patients with advanced soft tissue sarcomas: the ANNOUNCE randomized clinical trial. JAMA. (2020) 323(13):1266–76. doi: 10.1001/jama.2020.1707 PMC7139275 32259228

[16] Van Glabbeke M van Oosterom AT Oosterhuis JW Mouridsen H Crowther D Somers R. Prognostic factors for the outcome of chemotherapy in advanced soft tissue sarcoma: an analysis of 2,185 patients treated with anthracycline-containing first-line regimens–a European organization for research and treatment of cancer soft tissue and bone sarcoma group study. J Clin Oncol (1999) 17(1):150–7. doi: 10.1200/JCO.1999.17.1.150 10458228

[17] Younger E Husson O Asare B Benson C Judson I Miah A. Metastatic soft tissue sarcomas in adolescents and young adults: a specialist center experience. J Adolesc Young Adult Oncol (2020) 9(6):628–38. doi: 10.1089/jayao.2020.0010 PMC7757586 32379517

[18] Demetri GD von Mehren M Jones RL Hensley ML Schuetze SM Staddon A. Efficacy and safety of trabectedin or dacarbazine for metastatic liposarcoma or leiomyosarcoma after failure of conventional chemotherapy: results of a phase III randomized multicenter clinical trial. J Clin Oncol (2016) 34(8):786–93. doi: 10.1200/JCO.2015.62.4734 PMC5070559 26371143

[19] Judson I Verweij J Gelderblom H Hartmann JT Schoffski P Blay JY. Doxorubicin alone versus intensified doxorubicin plus ifosfamide for first-line treatment of advanced or metastatic soft-tissue sarcoma: a randomised controlled phase 3 trial. Lancet Oncol (2014) 15(4):415–23. doi: 10.1016/S1470-2045(14)70063-4 24618336

[20] van der Graaf WTA Blay J-Y Chawla SP Kim D-W Bui-Nguyen B Casali PG. Pazopanib for metastatic soft-tissue sarcoma (PALETTE): a randomised, double-blind, placebo-controlled phase 3 trial. Lancet (2012) 379(9829):1879–86. doi: 10.1016/S0140-6736(12)60651-5 22595799

[21] Bielack SS Kempf-Bielack B Delling G Exner GU Flege S Helmke K. Prognostic factors in high-grade osteosarcoma of the extremities or trunk: an analysis of 1,702 patients treated on neoadjuvant cooperative osteosarcoma study group protocols. J Clin Oncol (2002) 20(3):776–90. doi: 10.1200/JCO.2002.20.3.776 11821461

[22] Chisholm JC Marandet J Rey A Scopinaro M de Toledo JS Merks JH. Prognostic factors after relapse in nonmetastatic rhabdomyosarcoma: a nomogram to better define patients who can be salvaged with further therapy. J Clin Oncol (2011) 29(10):1319–25. doi: 10.1200/JCO.2010.32.1984 21357778

[23] Pasquali S Colombo C Pizzamiglio S Verderio P Callegaro D Stacchiotti S. High-risk soft tissue sarcomas treated with perioperative chemotherapy: improving prognostic classification in a randomised clinical trial. Eur J Cancer. (2018) 93:28–36. doi: 10.1016/j.ejca.2018.01.071 29475197

[24] Pasquali S Pizzamiglio S Touati N Litiere S Marreaud S Kasper B. The impact of chemotherapy on survival of patients with extremity and trunk wall soft tissue sarcoma: revisiting the results of the EORTC-STBSG 62931 randomised trial. Eur J Cancer. (2019) 109:51–60. doi: 10.1016/j.ejca.2018.12.009 30690293

[25] Hindi N Martin-Broto J. What is the standard indication of adjuvant or neoadjuvant chemotherapy in localized soft-tissue sarcoma? Curr Opin Oncol (2021) 33(4):329–35. doi: 10.1097/CCO.0000000000000742 33973551

[26] Brulard C Chibon F. Robust gene expression signature is not merely a significant p value. Eur J Cancer. (2013) 49(12):2771–3. doi: 10.1016/j.ejca.2013.03.033 23664094

[27] Chibon F Lagarde P Salas S Perot G Brouste V Tirode F. Validated prediction of clinical outcome in sarcomas and multiple types of cancer on the basis of a gene expression signature related to genome complexity. Nat Med (2010) 16(7):781–7. doi: 10.1038/nm.2174 20581836

[28] Chibon F Lesluyes T Valentin T Le Guellec S. CINSARC signature as a prognostic marker for clinical outcome in sarcomas and beyond. Genes Chromosomes Cancer. (2019) 58(2):124–9. doi: 10.1002/gcc.22703 30387235

[29] Bisogno G De Salvo GL Bergeron C Gallego Melcón S Merks JH Kelsey A. Vinorelbine and continuous low-dose cyclophosphamide as maintenance chemotherapy in patients with high-risk rhabdomyosarcoma (RMS 2005): a multicentre, open-label, randomised, phase 3 trial. Lancet Oncol (2019) 20(11):1566–75. doi: 10.1016/S1470-2045(19)30617-5 31562043

[30] Bielack SS Smeland S Whelan JS Marina N Jovic G Hook JM. Methotrexate, doxorubicin, and cisplatin (MAP) plus maintenance pegylated interferon Alfa-2b versus MAP alone in patients with resectable high-grade osteosarcoma and good histologic response to preoperative MAP: first results of the EURAMOS-1 good response randomized controlled trial. J Clin Oncol (2015) 33(20):2279–87. doi: 10.1200/JCO.2014.60.0734 PMC4486345 26033801

[31] Miettinen M. Immunohistochemistry of soft tissue tumours - review with emphasis on 10 markers. Histopathology. (2014) 64(1):101–18. doi: 10.1111/his.12298 PMC7670586 24111893

[32] Kumar S Perlman E Harris CA Raffeld M Tsokos M. Myogenin is a specific marker for rhabdomyosarcoma: an immunohistochemical study in paraffin-embedded tissues. Mod Pathol (2000) 13(9):988–93. doi: 10.1038/modpathol.3880179 11007039

[33] Goldstein MJ Mitchell EP. Carcinoembryonic antigen in the staging and follow-up of patients with colorectal cancer. Cancer Invest. (2005) 23(4):338–51. doi: 10.1081/CNV-58878 16100946

[34] Le DT Uram JN Wang H Bartlett BR Kemberling H Eyring AD. PD-1 blockade in tumors with mismatch-repair deficiency. N Engl J Med (2015) 372(26):2509–20. doi: 10.1056/NEJMoa1500596 PMC4481136 26028255

[35] Cercek A Lumish M Sinopoli J Weiss J Shia J Lamendola-Essel M. PD-1 blockade in mismatch repair-deficient, locally advanced rectal cancer. N Engl J Med (2022) 386(25):2363–76. doi: 10.1056/NEJMoa2201445 PMC9492301 35660797

[36] Blay JY Serrano C Heinrich MC Zalcberg J Bauer S Gelderblom H. Ripretinib in patients with advanced gastrointestinal stromal tumours (INVICTUS): a double-blind, randomised, placebo-controlled, phase 3 trial. Lancet Oncol (2020) 21(7):923–34. doi: 10.1016/S1470-2045(20)30168-6 PMC8383051 32511981

[37] Schoffski P Kubickova M Wozniak A Blay JY Strauss SJ Stacchiotti S. Long-term efficacy update of crizotinib in patients with advanced, inoperable inflammatory myofibroblastic tumour from EORTC trial 90101 CREATE. Eur J Cancer. (2021) 156:12–23. doi: 10.1016/j.ejca.2021.07.016 34392187

[38] Demetri GD von Mehren M Blanke CD Van den Abbeele AD Eisenberg B Roberts PJ. Efficacy and safety of imatinib mesylate in advanced gastrointestinal stromal tumors. N Engl J Med (2002) 347(7):472–80. doi: 10.1056/NEJMoa020461 12181401

[39] Demetri GD Reichardt P Kang YK Blay JY Rutkowski P Gelderblom H. (2013) Efficacy and safety of regorafenib for advanced gastrointestinal stromal tumours after failure of imatinib and sunitinib (GRID): an international, multicentre, randomised, placebo-controlled, phase 3 trial. Lancet (2013) 381(9863):295–302. doi: 10.1016/S0140-6736(12)61857-1 23177515 PMC3819942

[40] Roubaud G Kind M Coindre JM Maki RG Bui B Italiano A. Clinical activity of sorafenib in patients with advanced gastrointestinal stromal tumor bearing PDGFRA exon 18 mutation: a case series. Ann Oncol (2012) 23(3):804–5. doi: 10.1093/annonc/mdr631 22294526

[41] Bauer S Jones RL Blay JY Gelderblom H George S Schoffski P. Ripretinib versus sunitinib in patients with advanced gastrointestinal stromal tumor after treatment with imatinib (INTRIGUE): a randomized, open-label, phase III trial. J Clin Oncol (2022) 40(34):3918–28. doi: 10.1200/JCO.22.00294 PMC9746771 35947817

[42] Jones RL Serrano C von Mehren M George S Heinrich MC Kang YK. Avapritinib in unresectable or metastatic PDGFRA D842V-mutant gastrointestinal stromal tumours: long-term efficacy and safety data from the NAVIGATOR phase I trial. Eur J Cancer. (2021) 145:132–42. doi: 10.1016/j.ejca.2020.12.008 PMC9518931 33465704

[43] Judson I Morden JP Kilburn L Leahy M Benson C Bhadri V. Cediranib in patients with alveolar soft-part sarcoma (CASPS): a double-blind, placebo-controlled, randomised, phase 2 trial. Lancet Oncol (2019) 20(7):1023–34. doi: 10.1016/S1470-2045(19)30215-3 PMC6602919 31160249

[44] Italiano A Mir O Mathoulin-Pelissier S Penel N Piperno-Neumann S Bompas E. Cabozantinib in patients with advanced Ewing sarcoma or osteosarcoma (CABONE): a multicentre, single-arm, phase 2 trial. Lancet Oncol (2020) 21(3):446–55. doi: 10.1016/S1470-2045(19)30825-3 PMC8763616 32078813

[45] Cho WCS. Proteomics technologies and challenges. Genomics Proteomics Bioinf (2007) 5(2):77–85. doi: 10.1016/S1672-0229(07)60018-7 PMC5054093 17893073

[46] Network CGAR. Comprehensive and integrated genomic characterization of adult soft tissue sarcomas. Cell. (2017) 171(4):950–65.e28. 29100075 10.1016/j.cell.2017.10.014 PMC5693358

[47] Gounder MM Agaram NP Trabucco SE Robinson V Ferraro RA Millis SZ. Clinical genomic profiling in the management of patients with soft tissue and bone sarcoma. Nat Commun (2022) 13(1):3406. doi: 10.1038/s41467-022-30496-0 35705558 PMC9200814

[48] Nacev BA Sanchez-Vega F Smith SA Antonescu CR Rosenbaum E Shi H. Clinical sequencing of soft tissue and bone sarcomas delineates diverse genomic landscapes and potential therapeutic targets. Nat Commun (2022) 13(1):3405. doi: 10.1038/s41467-022-30453-x 35705560 PMC9200818

[49] Mertins P Mani DR Ruggles KV Gillette MA Clauser KR Wang P. Proteogenomics connects somatic mutations to signalling in breast cancer. Nature. (2016) 534(7605):55–62. doi: 10.1038/nature18003 27251275 PMC5102256

[50] Sinha A Huang V Livingstone J Wang J Fox NS Kurganovs N. The proteogenomic landscape of curable prostate cancer. Cancer Cell (2019) 35(3):414–27.e6. doi: 10.1016/j.ccell.2019.02.005 30889379 PMC6511374

[51] Zhang B Wang J Wang X Zhu J Liu Q Shi Z. Proteogenomic characterization of human colon and rectal cancer. Nature. (2014) 513(7518):382–7. doi: 10.1038/nature13438 PMC4249766 25043054

[52] Zhang H Liu T Zhang Z Payne SH Zhang B McDermott JE. Integrated proteogenomic characterization of human high-grade serous ovarian cancer. Cell. (2016) 166(3):755–65. doi: 10.1016/j.cell.2016.05.069 PMC4967013 27372738

[53] Wahjudi LW Bernhardt S Abnaof K Horak P Kreutzfeldt S Heining C. Integrating proteomics into precision oncology. Int J Cancer. (2021) 148(6):1438–51. doi: 10.1002/ijc.33301 32949162

[54] Boys EL Liu J Robinson PJ Reddel RR. Clinical applications of mass spectrometry-based proteomics in cancer: where are we? Proteomics (2022) 23:e2200238. doi: 10.1002/pmic.202200238 35968695

[55] Zhang B Whiteaker JR Hoofnagle AN Baird GS Rodland KD Paulovich AG. Clinical potential of mass spectrometry-based proteogenomics. Nat Rev Clin Oncol (2019) 16(4):256–68. doi: 10.1038/s41571-018-0135-7 PMC6448780 30487530

[56] Tully B Balleine RL Hains PG Zhong Q Reddel RR Robinson PJ. Addressing the challenges of high-throughput cancer tissue proteomics for clinical application: ProCan. Proteomics (2019) 19(21-22):e1900109. doi: 10.1002/pmic.201900109 31321850

[57] Macklin A Khan S Kislinger T. Recent advances in mass spectrometry based clinical proteomics: applications to cancer research. Clin Proteomics. (2020) 17:17. doi: 10.1186/s12014-020-09283-w 32489335 PMC7247207

[58] Poulos RC Hains PG Shah R Lucas N Xavier D Manda SS. Strategies to enable large-scale proteomics for reproducible research. Nat Commun (2020) 11(1):3793. doi: 10.1038/s41467-020-17641-3 32732981 PMC7393074

[59] Bordeaux J Welsh A Agarwal S Killiam E Baquero M Hanna J. Antibody validation. Biotechniques. (2010) 48(3):197–209. doi: 10.2144/000113382 20359301 PMC3891910

[60] Kondo T HS. Application of 2D-DIGE in cancer proteomics toward personalized medicine. In: He K, editor. Reverse chemical genetics. methods in molecular biology™. 577. Totowa, NJ: Humana Press (2009). p. 135–54. 10.1007/978-1-60761-232-2_11 19718514

[61] Begum H Murugesan P Tangutur AD. Western Blotting: a powerful staple in scientific and biomedical research. Biotechniques. (2022) 73(1):58–69. doi: 10.2144/btn-2022-0003 35775367

[62] Duraiyan J Govindarajan R Kaliyappan K Palanisamy M. Applications of immunohistochemistry. J Pharm Bioallied Sci (2012) 4(Suppl 2):S307–9. 10.4103/0975-7406.100281 PMC3467869 23066277

[63] Hall DA Ptacek J Snyder M. Protein microarray technology. Mech Ageing Dev (2007) 128(1):161–7. doi: 10.1016/j.mad.2006.11.021 PMC1828913 17126887

[64] Ludwig C Gillet L Rosenberger G Amon S Collins BC Aebersold R. Data-independent acquisition-based SWATH-MS for quantitative proteomics: a tutorial. Mol Syst Biol (2018) 14(8):e8126. doi: 10.15252/msb.20178126 30104418 PMC6088389

[65] Goncalves E Poulos RC Cai Z Barthorpe S Manda SS Lucas N. Pan-cancer proteomic map of 949 human cell lines. Cancer Cell (2022) 40(8):835–49.e8. doi: 10.1016/j.ccell.2022.06.010 35839778 PMC9387775

[66] Zhang B Yang L Wang X Fu D. Identification of a survival-related signature for sarcoma patients through integrated transcriptomic and proteomic profiling analyses. Gene. (2021) 764:145105. doi: 10.1016/j.gene.2020.145105 32882333

[67] Milighetti M Krasny L Lee ATJ Morani G Szecsei C Chen Y. Proteomic profiling of soft tissue sarcomas with SWATH mass spectrometry. J Proteomics. (2021) 241:104236. doi: 10.1016/j.jprot.2021.104236 33895336 PMC8135130

[68] Abeshouse A Adebamowo C Adebamowo SN Akbani R Akeredolu T Ally A. Comprehensive and integrated genomic characterization of adult soft tissue sarcomas. Cell. (2017) 171(4):950–65.e28. doi: 10.1016/j.cell.2017.10.014 29100075 PMC5693358

[69] Lou S Balluff B de Graaff MA Cleven AH Briaire-de Bruijn I Bovee JV. High-grade sarcoma diagnosis and prognosis: biomarker discovery by mass spectrometry imaging. Proteomics (2016) 16(11-12):1802–13. doi: 10.1002/pmic.201500514 27174013

[70] Kirik U Hansson K Krogh M Jonsson M Nilbert M James P. Discovery-based protein expression profiling identifies distinct subgroups and pathways in leiomyosarcomas. Mol Cancer Res (2014) 12(12):1729–39. doi: 10.1158/1541-7786.MCR-14-0072 25069693

[71] Yang J Eddy JA Pan Y Hategan A Tabus I Wang Y. Integrated proteomics and genomics analysis reveals a novel mesenchymal to epithelial reverting transition in leiomyosarcoma through regulation of slug. Mol Cell Proteomics. (2010) 9(11):2405–13. doi: 10.1074/mcp.M110.000240 PMC2984227 20651304

[72] Toulmonde M Lucchesi C Verbeke S Crombe A Adam J Geneste D. High throughput profiling of undifferentiated pleomorphic sarcomas identifies two main subgroups with distinct immune profile, clinical outcome and sensitivity to targeted therapies. EBioMedicine. (2020) 62:103131. doi: 10.1016/j.ebiom.2020.103131 33254023 PMC7708794

[73] Stewart E McEvoy J Wang H Chen X Honnell V Ocarz M. Identification of therapeutic targets in rhabdomyosarcoma through integrated genomic, epigenomic, and proteomic analyses. Cancer Cell (2018) 34(3):411–26.e19. doi: 10.1016/j.ccell.2018.07.012 30146332 PMC6158019

[74] Chaiyawat P Sungngam P Teeyakasem P Sirikaew N Klangjorhor J Settakorn J. Protein profiling of osteosarcoma tissue and soft callus unveils activation of the unfolded protein response pathway. Int J Oncol (2019) 54(5):1704–18. doi: 10.3892/ijo.2019.4737 PMC6438438 30816440

[75] Cheng DD Zhang HZ Yuan JQ Li SJ Yang QC Fan CY. Minichromosome maintenance protein 2 and 3 promote osteosarcoma progression *via* DHX9 and predict poor patient prognosis. Oncotarget. (2017) 8(16):26380–93. doi: 10.18632/oncotarget.15474 PMC5432265 28460433

[76] Kubota D Mukaihara K Yoshida A Tsuda H Kawai A Kondo T. Proteomics study of open biopsy samples identifies peroxiredoxin 2 as a predictive biomarker of response to induction chemotherapy in osteosarcoma. J Proteomics. (2013) 91:393–404. doi: 10.1016/j.jprot.2013.07.022 23911960

[77] Kikuta K Tochigi N Saito S Shimoda T Morioka H Toyama Y. Peroxiredoxin 2 as a chemotherapy responsiveness biomarker candidate in osteosarcoma revealed by proteomics. Proteomics Clin Appl (2010) 4(5):560–7. doi: 10.1002/prca.200900172 21137073

[78] Hang J Ouyang H Wei F Zhong Q Yuan W Jiang L. Proteomics and phosphoproteomics of chordoma biopsies reveal alterations in multiple pathways and aberrant kinases activities. Front Oncol (2022) 12:941046. doi: 10.3389/fonc.2022.941046 36248973 PMC9563620

[79] Shen Y Li M Xiong Y Gui S Bai J Zhang Y. Proteomics analysis identified ASNS as a novel biomarker for predicting recurrence of skull base chordoma. Front Oncol (2021) 11:698497. doi: 10.3389/fonc.2021.698497 34540668 PMC8440958

[80] Zhou H Chen CB Lan J Liu C Liu XG Jiang L. Differential proteomic profiling of chordomas and analysis of prognostic factors. J Surg Oncol (2010) 102(7):720–7. doi: 10.1002/jso.21674 20721957

[81] Kikuta K Tochigi N Shimoda T Yabe H Morioka H Toyama Y. Nucleophosmin as a candidate prognostic biomarker of ewing's sarcoma revealed by proteomics. Clin Cancer Res (2009) 15(8):2885–94. doi: 10.1158/1078-0432.CCR-08-1913 19351769

[82] Liu Y Li Z Xu Z Jin X Gong Y Xia X. Proteomic maps of human gastrointestinal stromal tumor subgroups. Mol Cell Proteomics. (2019) 18(5):923–35. doi: 10.1074/mcp.RA119.001361 PMC6495251 30804049

[83] Atay S Wilkey DW Milhem M Merchant M Godwin AK. Insights into the proteome of gastrointestinal stromal tumors-derived exosomes reveals new potential diagnostic biomarkers. Mol Cell Proteomics. (2018) 17(3):495–515. doi: 10.1074/mcp.RA117.000267 29242380 PMC5836374

[84] Ichikawa H Yoshida A Kanda T Kosugi S Ishikawa T Hanyu T. Prognostic significance of promyelocytic leukemia expression in gastrointestinal stromal tumor; integrated proteomic and transcriptomic analysis. Cancer Sci (2015) 106(1):115–24. doi: 10.1111/cas.12565 PMC4317774 25457157

[85] Kikuta K Kubota D Saito T Orita H Yoshida A Tsuda H. Clinical proteomics identified ATP-dependent RNA helicase DDX39 as a novel biomarker to predict poor prognosis of patients with gastrointestinal stromal tumor. J Proteomics. (2012) 75(4):1089–98. doi: 10.1016/j.jprot.2011.10.005 22119546

[86] Da Riva L Bozzi F Mondellini P Micciche F Fumagalli E Vaghi E. Proteomic detection of a large amount of SCGFalpha in the stroma of GISTs after imatinib therapy. J Transl Med (2011) 9:158. doi: 10.1186/1479-5876-9-158 21943129 PMC3192683

[87] Suehara Y Kondo T Seki K Shibata T Fujii K Gotoh M. Pfetin as a prognostic biomarker of gastrointestinal stromal tumors revealed by proteomics. Clin Cancer Res (2008) 14(6):1707–17. doi: 10.1158/1078-0432.CCR-07-1478 18347171

[88] Klemen ND Hwang S Bradic M Rosenbaum E Dickson MA Gounder MM. Long-term follow-up and patterns of response, progression, and hyperprogression in patients after PD-1 blockade in advanced sarcoma. Clin Cancer Res (2022) 28(5):939–47. doi: 10.1158/1078-0432.CCR-21-3445 PMC8898277 34965948

[89] Burgess MA Bolejack V Schuetze S Van Tine BA Attia S Riedel RF. Clinical activity of pembrolizumab (P) in undifferentiated pleomorphic sarcoma (UPS) and dedifferentiated/pleomorphic liposarcoma (LPS): final results of SARC028 expansion cohorts. J Clin Oncol (2019) 37(15_suppl):11015–. doi: 10.1200/JCO.2019.37.15_suppl.11015

[90] Lazcano R Barreto CM Salazar R Carapeto F Traweek RS Leung CH. The immune landscape of undifferentiated pleomorphic sarcoma. Front Oncol (2022) 12. doi: 10.3389/fonc.2022.1008484 PMC9597628 36313661

[91] Leiner J Le Loarer F. The current landscape of rhabdomyosarcomas: an update. Virchows Arch (2020) 476(1):97–108. doi: 10.1007/s00428-019-02676-9 31696361

[92] Beck AH Lee CH Witten DM Gleason BC Edris B Espinosa I. Discovery of molecular subtypes in leiomyosarcoma through integrative molecular profiling. Oncogene. (2010) 29(6):845–54. doi: 10.1038/onc.2009.381 PMC2820592 19901961

[93] Anderson ND Babichev Y Fuligni F Comitani F Layeghifard M Venier RE. Lineage-defined leiomyosarcoma subtypes emerge years before diagnosis and determine patient survival. Nat Commun (2021) 12(1):4496. doi: 10.1038/s41467-021-24677-6 34301934 PMC8302638

[94] Guo X Jo VY Mills AM Zhu SX Lee CH Espinosa I. Clinically relevant molecular subtypes in leiomyosarcoma. Clin Cancer Res (2015) 21(15):3501–11. doi: 10.1158/1078-0432.CCR-14-3141 PMC4526352 25896974

[95] Chudasama P Mughal SS Sanders MA Hubschmann D Chung I Deeg KI. Integrative genomic and transcriptomic analysis of leiomyosarcoma. Nat Commun (2018) 9(1):144. doi: 10.1038/s41467-017-02602-0 29321523 PMC5762758

[96] Blay JY Kang YK Nishida T von Mehren M. Gastrointestinal stromal tumours. Nat Rev Dis Primers. (2021) 7(1):22. doi: 10.1038/s41572-021-00254-5 33737510

[97] Martin-Broto J Gutierrez A Garcia-Del-Muro X Lopez-Guerrero JA Martinez-Trufero J de Sande LM. Prognostic time dependence of deletions affecting codons 557 and/or 558 of KIT gene for relapse-free survival (RFS) in localized GIST: a Spanish group for sarcoma research (GEIS) study. Ann Oncol (2010) 21(7):1552–7. doi: 10.1093/annonc/mdq047 20231303

[98] Kondo T Suehara Y Kikuta K Kubota D Tajima T Mukaihara K. Proteomic approach toward personalized sarcoma treatment: lessons from prognostic biomarker discovery in gastrointestinal stromal tumor. Proteomics Clin Appl (2013) 7(1-2):70–8. doi: 10.1002/prca.201200085 23281253

[99] Kubota D Orita H Yoshida A Gotoh M Kanda T Tsuda H. Pfetin as a prognostic biomarker for gastrointestinal stromal tumor: validation study in multiple clinical facilities. Jpn J Clin Oncol (2011) 41(10):1194–202. doi: 10.1093/jjco/hyr121 21903705

[100] Li J Lu Y Akbani R Ju Z Roebuck PL Liu W. TCPA: a resource for cancer functional proteomics data. Nat Methods (2013) 10(11):1046–7. doi: 10.1038/nmeth.2650 PMC4076789 24037243

[101] Frolov A Chahwan S Ochs M Arnoletti JP Pan ZZ Favorova O. Response markers and the molecular mechanisms of action of gleevec in gastrointestinal stromal tumors. Mol Cancer Ther (2003) 2(8):699–709. 12939459

[102] Klaeger S Heinzlmeir S Wilhelm M Polzer H Vick B Koenig PA. The target landscape of clinical kinase drugs. Science (2017) 358(6367):eaan4368. doi: 10.1126/science.aan4368 29191878 PMC6542668

[103] Fordham AM Ekert PG Fleuren EDG. Precision medicine and phosphoproteomics for the identification of novel targeted therapeutic avenues in sarcomas. Biochim Biophys Acta Rev Cancer. (2021) 1876(2):188613. doi: 10.1016/j.bbcan.2021.188613 34390800

[104] Wiechmann S Ruprecht B Siekmann T Zheng R Frejno M Kunold E. Chemical phosphoproteomics sheds new light on the targets and modes of action of AKT inhibitors. ACS Chem Biol (2021) 16(4):631–41. doi: 10.1021/acschembio.0c00872 33755436

